# Editorial: Frailty and oxidative stress

**DOI:** 10.3389/fragi.2023.1345486

**Published:** 2024-03-06

**Authors:** Pasquale Mone, Antonio De Luca, Gaetano Santulli

**Affiliations:** ^1^ Department of Medicine and Health Sciences “Vincenzo Tiberio”, University of Molise, Campobasso, Italy; ^2^ Albert Einstein College of Medicine, New York, NY, United States; ^3^ Department of Mental and Physical Health and Preventive Medicine, Campania University “Luigi Vanvitelli”, Caserta, Italy; ^4^ International Translational Research and Medical Education (ITME) Consortium, Department of Advanced Biomedical Sciences, “Federico II” University, Naples, Italy

**Keywords:** frailty, older adults, comorbidities, oxidative stress, SGLT2 inhibitors

Frailty is a prevailing condition in older adults, increasing the risk of death, hospitalizations, and adverse outcomes, while driving both cognitive and physical impairment. With the increase in life expectancy, aging is steeply rising, leading to frailty with a higher risk of losing independence (Ferrucci and Fabbri, 2018).

Aging is known to be associated with oxidative stress linked to reactive oxygen and nitrogen species (Liguori et al., 2018).

The present Research Topic has been focused on “*Frailty and oxidative stress*.” In particular, Tong et al. reviewed the functional role of mitochondrial dynamics in retinal pigment epithelial aging and degeneration. An overview of the literature on the mechanistic roles of miRNA-34a in diabetes and frailty evidenced that this miRNA could be involved in diabetes-induced endothelial dysfunction (Mone et al.). Wei et al. systematically reviewed the role of GLP-1 receptor agonists in arrhythmias in diabetic patients.

In an original paper, Song et al. correlated the severity of non-alcoholic fatty liver disease with cardiovascular outcomes in pre-hypertension and hypertension. In another elegant study, Salis et al. focused on frail elders with atrial fibrillation showing a worst frailty in presence of more comorbidities. Last but not least, Qi et al. published a national cross sectional study social frailty in Chinese older adults with cardiovascular and cerebrovascular diseases.

Based on the published papers, we can conclude that frail patients have many comorbidities, including cardiovascular and metabolic diseases (e.g., hypertension, heart failure, diabetes, and metabolic syndrome), which further increase oxidative stress. In this context, managing frail patients represents a great challenge since there is no accepted treatment for oxidative stress in frail older adults with comorbidities (Mone et al., 2022b). Empagliflozin, a sodium-glucose cotransporter-2 inhibitor [SGLT2-inhibitor (Varzideh et al., 2021)], is usually prescribed in diabetic patients and drives strong effects on heart failure, chronic kidney disease, and atherosclerotic cardiovascular disease (ElSayed et al., 2023; Forzano et al., 2023). In a previous investigation, we evidenced the positive effects of Empagliflozin on frailty in hypertensive and diabetic elders, evidencing a significant result on oxidative stress in human endothelial cells (Mone et al., 2022c). Intriguingly, we also observed beneficial effects on endothelial micro-RNAs in frail diabetic elders with heart failure and preserved ejection fraction (Mone et al., 2023). Therefore, based on their antioxidative effects in frailty, SGLT2 inhibitors should be endorsed as anti-frailty drugs (Santulli et al., 2023).
